# Lymphocyte Inhibition Mechanisms and Immune Checkpoints in COVID-19: Insights into Prognostic Markers and Disease Severity

**DOI:** 10.3390/medicina61020189

**Published:** 2025-01-22

**Authors:** Martina Schniederova, Anna Bobcakova, Marian Grendar, Adam Markocsy, Andrej Ceres, Michal Cibulka, Dusan Dobrota, Milos Jesenak

**Affiliations:** 1 Institute of Clinical Immunology and Medical Genetics, Jessenius Faculty of Medicine in Martin, Comenius University in Bratislava, Martin University Hospital, 03659 Martin, Slovakia; martina.schniederova@unm.sk (M.S.); anna.bobcakova@jfmed.uniba.sk (A.B.); adam.markocsy@unm.sk (A.M.); michal.cibulka@uniba.sk (M.C.); 2 Department of Pulmonology and Phthisiology, Jessenius Faculty of Medicine in Martin, Comenius University in Bratislava, Martin University Hospital, 03659 Martin, Slovakia; 3 Department of Paediatrics and Adolescent Medicine, Jessenius Faculty of Medicine in Martin, Comenius University in Bratislava, Martin University Hospital, 03659 Martin, Slovakia; 4 Biomed—Centre for Biomedicine, Jessenius Faculty of Medicine in Martin, Comenius University in Bratislava, 03659 Martin, Slovakia; 5 Department of Medical Biochemistry, Jessenius Faculty of Medicine in Martin, Comenius University in Bratislava, 03659 Martin, Slovakia; dusan.dobrota@uniba.sk; 6 Department of Clinical Biochemisty, Jessenius Faculty of Medicine in Martin, Comenius University in Bratislava, Martin University Hospital, 03659 Martin, Slovakia

**Keywords:** immune checkpoint inhibitors, immune predictors, COVID-19, machine-learning algorithm

## Abstract

*Background and Objectives*: Immune checkpoint inhibitors such as PD-1 and TIM-3 play an important role in regulating the host immune response and are proposed as potential prognostic markers and therapeutic targets in severe cases of COVID-19. We evaluated the expression of PD-1 and TIM-3 on T cells, as well as the concentration of sPD-1 in plasma, to clarify the role of these molecules in patients infected with SARS-CoV-2. *Materials and Methods:* In this retrospective observational study, we analysed the expression of PD-1 and TIM-3 on CD4^+^ and CD8^+^ T cells upon admission and after 7 days of hospitalisation in 770 adult patients. We also evaluated sPD-1 levels in the plasma of 145 patients at different stages of COVID-19 and of 11 control subjects. Molecules were determined using conventional flow cytometry and ELISA and the data were statistically processed. *Results:* We observed a significantly higher expression of PD-1 on CD4^+^ cells in deceased patients than in those with mild-to-moderate disease. All patients with COVID-19 exhibited a significantly higher expression of TIM-3 on both CD4^+^ and CD8^+^ T cells compared to controls. After 1 week of hospitalisation, there was no significant change in PD-1 or TIM-3 expression on CD4^+^ or CD8^+^ T cells across the studied groups. sPD-1 concentrations were not significantly different between survivors and non-survivors. Plasma sPD-1 levels did not correlate with PD-1 expression on T cells, but a significant correlation was observed between CD4^+^ PD-1 and CD8^+^ PD-1. Using machine-learning algorithms, we supported our observations and confirmed immunological variables capable of predicting survival, with AUC = 0.786. *Conclusions:* Analysis of the immune response may be useful for monitoring and predicting the course of COVID-19 upon admission. However, it is essential to evaluate complex immune parameters in conjunction with other key clinical and laboratory indicators.

## 1. Introduction

The infection caused by the novel coronavirus [coronavirus disease 2019 (COVID-19)] became a global pandemic lasting more than 3 years and had an unprecedented impact on the quality of daily life. The immune response in patients with a critical-to-fatal disease course comprises three stages: a normal or hypofunctional immune response, excessive activation, and a state of immune anergy and/or exhaustion [1]. As a result of excessive activation and antigenic stimulation of T-cell receptors, exhaustion of T cells occurs due to robust co-inhibitory molecular signalling or the limited presence of co-stimulatory receptors such as CD28. Exhausted T cells express co-inhibitory receptors on their membranes, such as programmed cell death receptor 1 (PD-1), T-cell immunoglobulin and mucin-domain containing-3 (TIM-3), cytotoxic T lymphocyte protein 4 (CTLA-4), and lymphocyte activation gene 3 (LAG-3). The expression of these receptors increases significantly during the progression from asymptomatic to symptomatic stages and potentially from mild-to-critical stages of infection [2]. A severe course of COVID-19 displays dysregulated expression of checkpoint molecules, mainly PD-1 and its ligand PD-L1, as well as TIM-3, suggesting these checkpoint molecules could serve as prognostic markers and therapeutic targets in severe cases of COVID-19 [3].

Monitoring selected immune parameters could help in understanding the dynamics of immune system activation and in identifying suitable prognostic markers with regard to the clinical progression of the disease and unforeseen complications. However, despite considerable effort, it remains unclear whether the observed changes in immune parameters are a direct consequence of COVID-19 or predisposing factors in the development of the disease and its critical course. Several questions regarding the regulation and dynamics of the immune response remain unanswered and should be the subject of future research [4].

## 2. Materials and Methods

In this single-centre retrospective observational study, we analysed the expression of immune checkpoint inhibitors PD-1 and TIM-3 on CD4^+^ and CD8^+^ T cells upon admission and after 7 days of hospitalisation in 770 adult patients with COVID-19 at Martin University Hospital between March 2020 and August 2021. We evaluated the level of soluble PD-1 in the plasma of 145 patients at different stages of COVID-19 infection (Groups A, B, C, and D) and of 11 control subjects. For a comprehensive view of the immune system, we also analysed the expression and absolute number of lymphocyte subsets (CD3^+^, CD4^+^, CD8^+^, CD19^+^, and NK), the immunoregulatory index (IRI, calculated as the CD4^+^/CD8^+^ ratio), and the absolute number of leucocytes and lymphocytes. Each parameter was analysed in relation to disease course, healthy controls, and survivor status.

### 2.1. Study Participants (Basic Characteristics)

In total, 770 participants unaware of previous exposure to severe acute respiratory syndrome coronavirus 2 (SARS-CoV-2) met the inclusion criteria, namely, a laboratory-confirmed SARS-CoV-2 infection by quantitative reverse transcription polymerase chain reaction from a nasopharyngeal swab or antigen test along with typical symptoms. The patients were divided into four groups according to severity of COVID-19 infection [5]. Group A (n = 100) comprised patients with a mild-to-moderate course of COVID-19 (without the need for oxygen supplementation). Group B (n = 378) comprised patients with severe COVID-19 (bilateral pneumonia with hypoxemic respiratory failure). Group C (n = 90) comprised patients with a critical course of COVID-19 (hospitalised in the intensive care unit with invasive or non-invasive ventilation support). Deceased patients were assigned to Group D (n = 201), median time to death from admission to the hospital in group D was 10 days with interquartile range (IQR 7, 15). Recruitment exclusion criteria included participants who disagreed to take part in our biomedical research study.

The control cohort (Group K) comprised 30 blood donors from the National Blood Transfusion Service in Martin (January 2022). Only healthy controls without previously confirmed COVID-19 and at least 2 weeks after vaccination were included. The characteristics of the participants are shown in Table 1 and Table 2.

The study was approved by the local ethics committee (decision no. EK UNM 77/2020, dated 15 June 2020; EK JLF UK 74/2021, dated 16 December 2021).

### 2.2. Multiparametric Flow Cytometry

**Figure 1:**
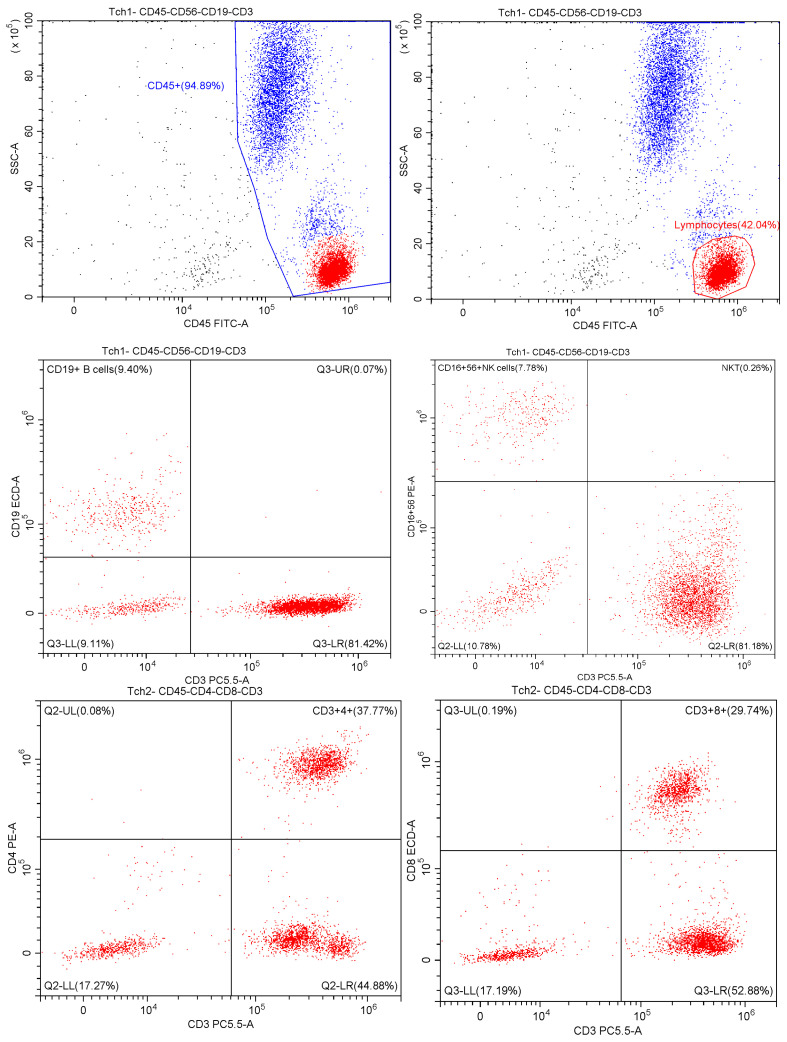
The hierarchical gating strategy used for immunophenotyping immune cells in patients with COVID-19. The immune cells were labelled with a multi-coloured combination of monoclonal antibodies analysed in a flow cytometer, and evaluated based on positivity for a given characteristic.

Blood samples were collected in BD Vacutainer^®^ tubes (Becton, Dickinson, and Company, Franklin Lakes, NJ, USA). For immunophenotyping, we used the fluorescently labelled monoclonal antibodies tetraCHROME 1 (CD45-FITC/CD56-RD1/CD19-ECD/CD3-PC5), tetraCHROME 2 (CD45-FITC/CD4-RD1/CD8-ECD/CD3-PC5), CD279-PC7 (PD-1), and CD366-PE/Cy7 (TIM-3) (Beckman Coulter, Brea, CA, USA; SONY Biotechnology, San Jose, CA, USA) at a volume of 5 µL, to which we added 50 µL of whole blood. Specific isotype controls were used as negative controls. After 30 min of incubation in the dark at room temperature, the cell suspensions were lysed with 500 µL of lysis solution and incubated for 10 min in the dark at room temperature. Analysis was performed using CytExpert software version 2.0.2.18 and a DxFLEX flow cytometer (Beckman Coulter). Peripheral mononuclear cells were defined by forward scattering and side scattering. Using manual gating, we defined the population of all haematopoietic cells expressing the CD45^+^ marker. Subsequently, we identified populations of T cells (CD45^+^CD3^+^), B lymphocytes (CD45^+^CD19^+^), and NK cells (CD45^+^CD3^−^CD16^+^CD56^+^). Within the T cells, we determined subpopulations of cytotoxic T cells (CD3^+^CD8^+^) and helper T cells (CD3^+^CD4^+^) (Figure 1).

**Figure 2:**
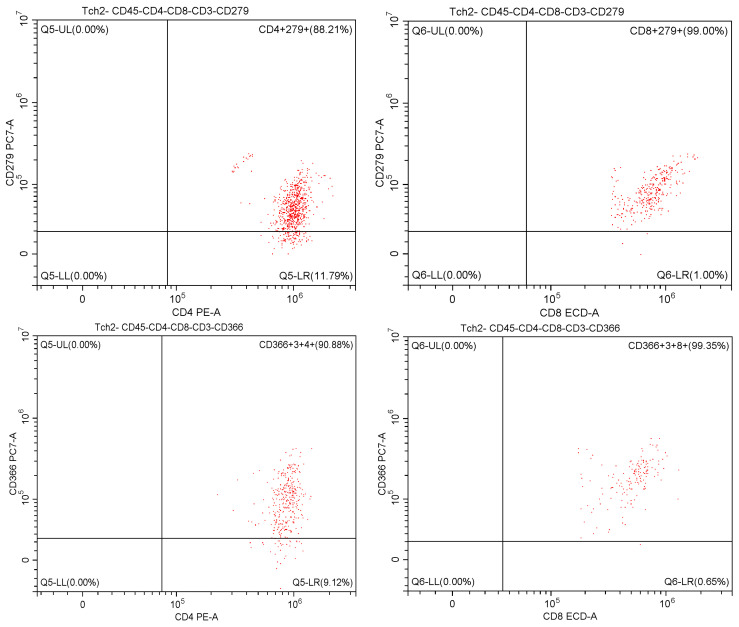
Immunophenotypic analysis of immune inhibitory receptors on T lymphocytes. The population of cells that express CD4^+^ and CD8^+^ surface inhibitory receptors was visualized by separate multicolour labelling for panels CD3^+^CD4^+^CD8^+^CD279^+^ and CD3^+^CD4^+^CD8^+^CD366^+^.

Exhausted T cell populations were defined based on the following phenotypes: CD3^+^CD4^+^CD279^+^ for helper T cells, CD3^+^CD8^+^CD279^+^ for cytotoxic T cells, and CD3^+^CD4^+^CD366^+^ and CD3^+^CD8^+^CD366^+^ for TIM-3 expression (Figure 2).

### 2.3. Enzyme-Linked Immunosorbent Assay (ELISA)

Soluble PD-1 in plasma was determined from frozen plasma samples (−80 °C) using the Human PD-1 ELISA Kit (ab252360) (Abcam, Cambridge, UK) according to the manufacturer’s protocol. We evaluated the level of soluble PD-1 in the plasma of 145 patients at different stages of COVID-19 infection (Groups A, B, C, and D) and of 11 control subjects. A detailed description of the participants is provided in Table 3.

### 2.4. Data Analysis

Data were explored and analysed using R [R Core Team (2021); R: a language and environment for statistical computing; R Foundation for Statistical Computing, Vienna, Austria. URL: https://www.R-project.org/, ver. 4.0.5 (accessed on 31 March 2021)]. Continuous variables were summarised as mean, standard deviation, and median. Associations between variables were visualised using a pairs plot. The multivariate linear regression model was used to model the association between an immunological variable and age, gender, length of hospital stay and group. Box–Cox transformation was applied to transform the response variable closer to normality. Based on the fitted multivariate regression model, marginal means and post hoc tests were performed using the obtain estimated marginal means (EMMs) library, and the estimates were back-transformed. The estimated marginal means, together with their 95% confidence intervals, were visualised using a marginal means plot. For immunological variables measured at two points in time (time 0 and time 1), difference (dif = time 0 − time 1) was computed and summarised by mean, median, and standard deviation. The distribution of differences was explored using a histogram, density plot, and boxplot, and overlaid with a swarm plot. Median regression was used to model associations between the difference in immunological variables and age, gender, length of hospital stay, and group (A, B, C, and D). Based on the fitted model, marginal means were computed and post hoc pairwise comparisons were performed using the Benjamini–Hochberg adjustment of *p* values. An imbalanced random forest (RF) machine-learning algorithm was trained to predict survivors (Groups A, B, and C) versus non-survivors (Group D) using immunological variables as predictors. Predictors were ranked by variable importance (VIMP) as well as graph depth. A receiver operating characteristic (ROC) curve, based on out-of-bag data, was used to quantify predictive power. Spearman’s correlation coefficient was used with logarithmic transformation applied to the data. The correlation coefficient between the level of soluble PD-1 (sPD-1) and laboratory parameters was calculated using Spearman’s rank correlation coefficient. Spearman’s rho (ρ) and *p* values were calculated as well.

## 3. Results

### 3.1. Expression of PD-1 and TIM-3 in Patients with COVID-19 upon Hospital Admission

**Figure 3:**
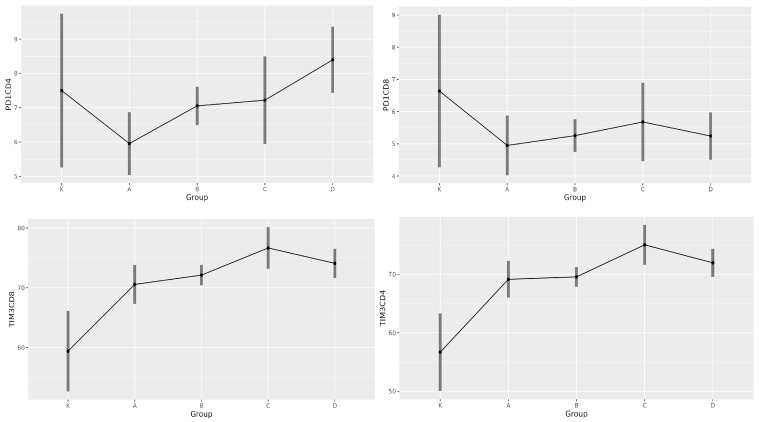
Marginal means plots showing the estimated marginal means (back-transformed) for the percentage of inhibitory molecules PD-1 and TIM-3 upon hospital admission.

We observed a significantly higher expression of PD-1 on CD4^+^ cells upon hospital admission in deceased patients (Group D) than in patients with mild-to-moderate disease (Group A). There was no significant difference in PD-1 expression on CD8^+^ cells between groups. All COVID-19 groups (A, B, C, and D) showed a significantly higher expression of TIM-3 on both CD4^+^ and CD8^+^ T cells than the controls (Figure 3, Table 4). Immunological parameters, including lymphocyte subsets (CD3^+^, CD4^+^, CD8^+^, CD19^+^, and NK), the IRI (CD4^+^/CD8^+^), and the absolute number of leucocytes and lymphocytes, correlated with severity of infection and were summarised by mean, standard deviation, and median in an exploratory data analysis (Table S1).

Post hoc pairwise comparisons revealed statistically significant differences in inhibitor expression between Groups A and D for PD-1 on CD4^+^ cells and between the control group and COVID-19 groups for TIM-3 (Table 5).

Similarly, comparisons between COVID-19 survivors (Groups A, B, and C) and non-survivors (Group D) revealed a significantly lower expression of PD-1 on CD4^+^ T cells (*p* = 0.007) in survivors. No significant difference was found in PD-1 expression on CD8^+^ T cells between survivors and non-survivors. Although the differences in TIM-3 expression on CD4^+^ and CD8^+^ T cells were not statistically significant, a mild trend toward higher expression was observed in non-survivors. Immunological parameters (CD3^+^, CD4^+^, CD8^+^, CD19^+^, and NK), the IRI (CD4^+^/CD8^+^), and the absolute number of leucocytes and lymphocytes, summarised by mean, standard deviation, and median, between survivors (Groups A, B, C) and non-survivors (Group D) upon admission to the hospital are listed in the Supplementary Material (Table S2).

**Figure 4:**
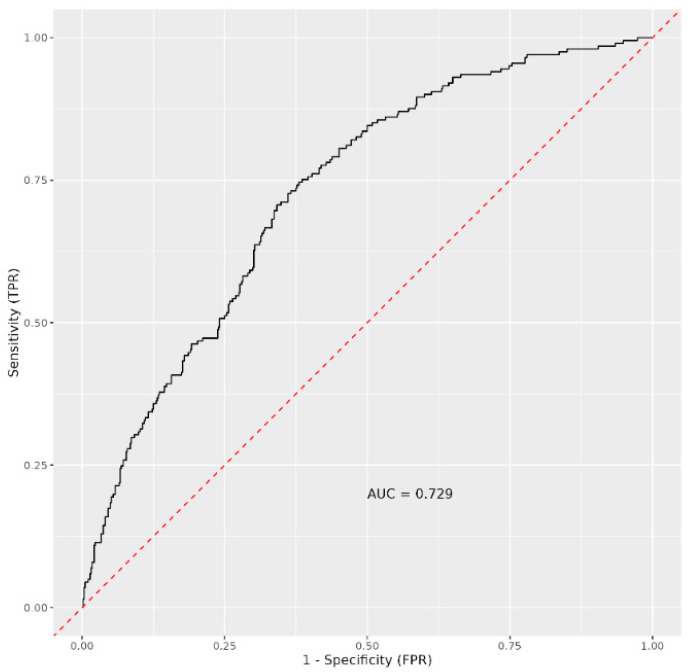
ROC curve with AUC values of all immune parameters between survivors and non-survivors.

We also determined the area under the curve (AUC) value for all immune parameters (leucocytes/nL, lymphocytes/nL, CD3^+^ %, CD3^+^ cells/µL, CD19^+^ %, CD19^+^ cells/µL, CD4^+^ %, CD4^+^ cells/µL, CD8^+^ %, CD8^+^ cells/µL, NK %, NK cells/µL, IRI, PD-1 CD4^+^ %, PD-1 CD8^+^ %, TIM-3 CD4^+^ %, and TIM-3 CD8^+^ %). A 70% sensitivity and 70% specificity for predicting COVID-19 mortality was captured, with an AUC value of 0.729 (Figure 4).

### 3.2. Expression of PD-1 and TIM-3 in Patients with COVID-19 After 1 Week

We also evaluated the changes in inhibitory molecules during the first week of hospitalisation. After 1 week, there was no significant change in the expression of PD-1 or TIM-3 on CD4^+^ and CD8^+^ T cells across the studied groups. For the immunological variables measured at two points in time—upon hospital admission (time 0) and after 1 week of hospitalisation (time 1)—the difference (dif = time 0 − time 1) was calculated and summarised by mean, standard deviation, and median. Patient characteristics are also provided in Table S3. In general, we observed a significant increase in the counts of all basic lymphocyte subsets during the first week of hospitalisation, except in NK cells, which significantly decreased in non-survivors compared to survivors.

**Figure 5:**
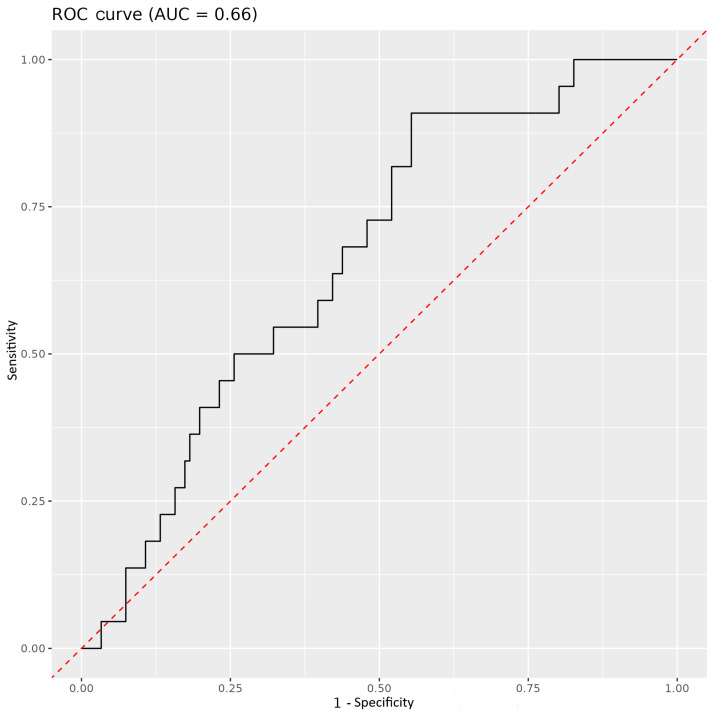
An ROC curve showing AUC values derived from the difference between time 0 and time 1 for all immune parameters, comparing survivors and non-survivors.

The AUC value (AUC = 0.66), derived from the difference between time 0 and time 1, for all immune parameters (leucocytes/nL, lymphocytes/nL, CD3^+^ %, CD3^+^ cells/µL, CD19^+^ %, CD19^+^ cells/µL, CD4^+^ %, CD4^+^ cells/µL, CD8^+^ %, CD8^+^ cells/µL, NK %, NK cells/µL, IRI, PD-1 CD4^+^ %, PD-1 CD8^+^ %, TIM-3 CD4^+^ %, and TIM-3 CD8^+^ %) did not provide a reliable discriminating factor for stratifying survivors and non-survivors based on the difference (Figure 5).

### 3.3. Immune Parameters as Predictors of COVID-19 Outcome

**Figure 6:**
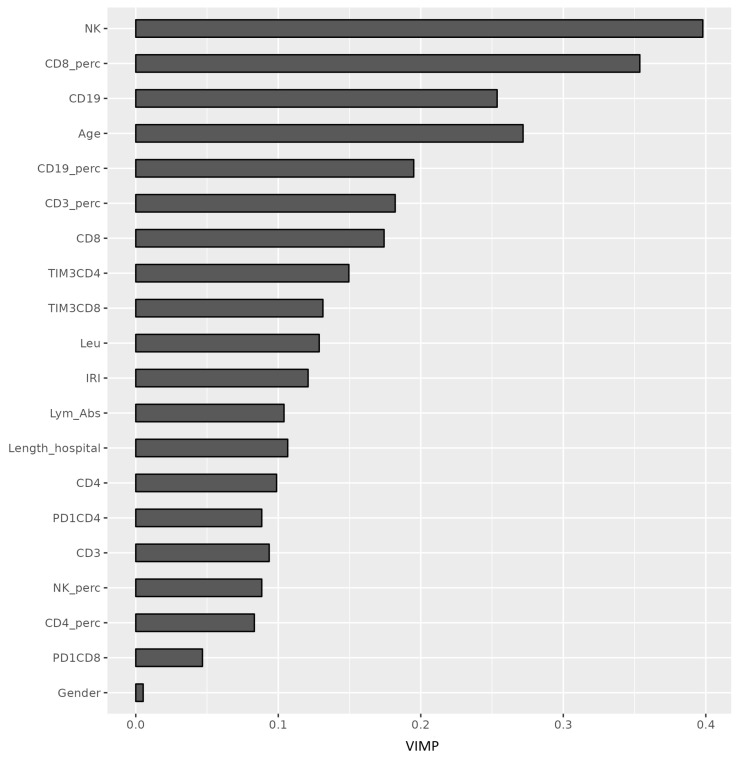
Plot depicting the ranking of predictors and confounders by variable importance, VIMP (*x*-axis).

**Figure 7:**
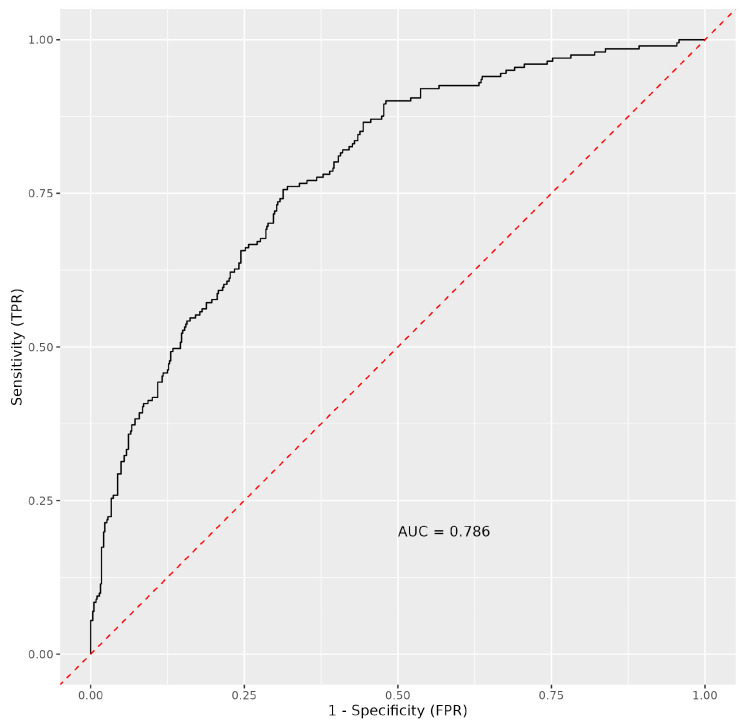
ROC curve with AUC values for immunological variables and confounders (age, gender, and length of hospital stay) in order to predict survival status in COVID-19 infections.

An imbalanced RF machine-learning algorithm was trained on the study’s immunological data to predict survivors versus non-survivors using immunological variables as predictors (Figure 4). Predictors were ranked based on VIMP, minimal graph depth, and elastic net. In this analysis, in addition to examining absolute counts of parameters, we also focused on percentages of individual subsets and absolute counts of leucocytes and lymphocytes. These parameters, which are part of conventional flow cytometry, provide a comprehensive view of a patient’s immune response. Variable importance was determined in all examined variables to be positive, whereas the most important were the absolute number of NK cells and the percentage of CD8^+^ and CD19^+^ T lymphocytes. Among the confounders, the age composition of individuals turned out to be the most important (Figure 6). Selected variables can be potentially helpful to distinguish between survivors and non-survivors with a predictive ability of AUC = 0.786 (Figure 7).

### 3.4. Soluble PD-1 as a Predictor of COVID-19

**Figure 8:**
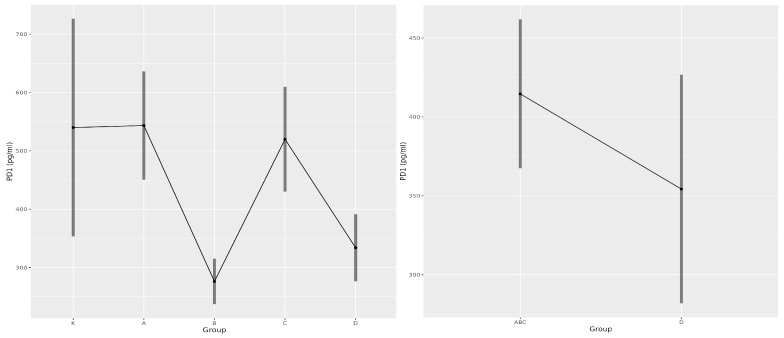
Marginal means plots for the concentration of sPD-1 across groups and between survivors and non-survivors.

While similar levels of sPD-1 were detected in the control group, Group A, and Group C, lower concentrations were measured in Group B and non-survivors. Statistically significant differences were observed between Groups A and B (*p* = 0.000), A and D (*p* = 0.002), B and C (*p* = 0.000), and C and D (*p* = 0.006). When comparing survivors (Groups A, B, C) with non-survivors (Group D), no statistically significant difference was observed (Figure 8). The marginal means of the sPD-1 concentration in all analysed groups are presented in Table 6. A detailed analysis of sPD-1 is provided in the Supplementary Material (Table S4).

**Figure 9:**
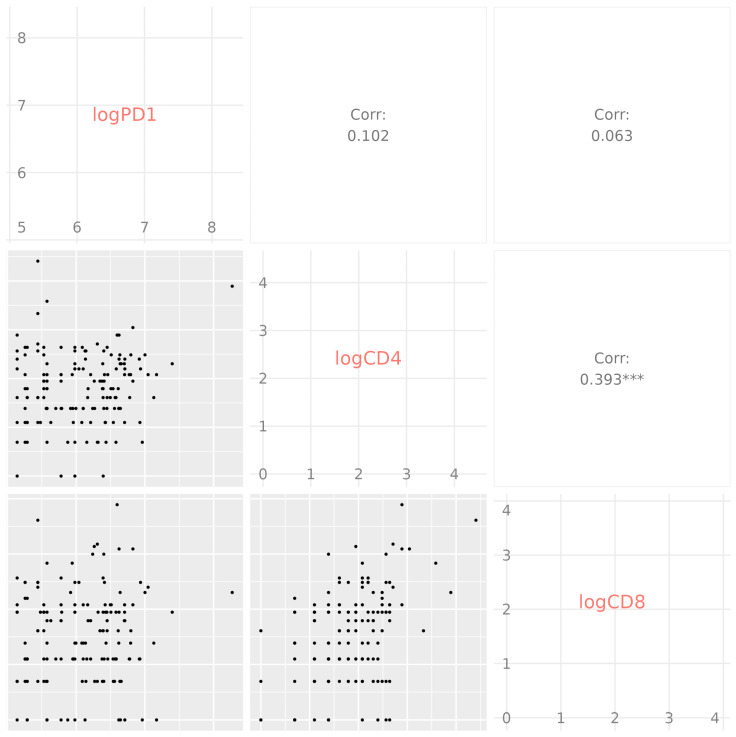
Spearman correlation between sPD-1, CD4^+^ PD-1, and CD8^+^ PD-1 for all analysed COVID-19 groups combined and control subjects. A positive correlation was only observed between CD4^+^ PD-1 and CD8^+^ PD-1 (*p* < 0.001). *** *p* < 0.001.

We also examined the correlation between the concentration of sPD-1 and the expression of membrane-bound PD-1 on CD4^+^ and CD8^+^ T cells across all COVID-19 groups and in comparison with the control group (Figure 9). A positive correlation was only found between the expression of PD-1 on CD4^+^ and CD8^+^ T cells (*p* < 0.001).

**Figure 10:**
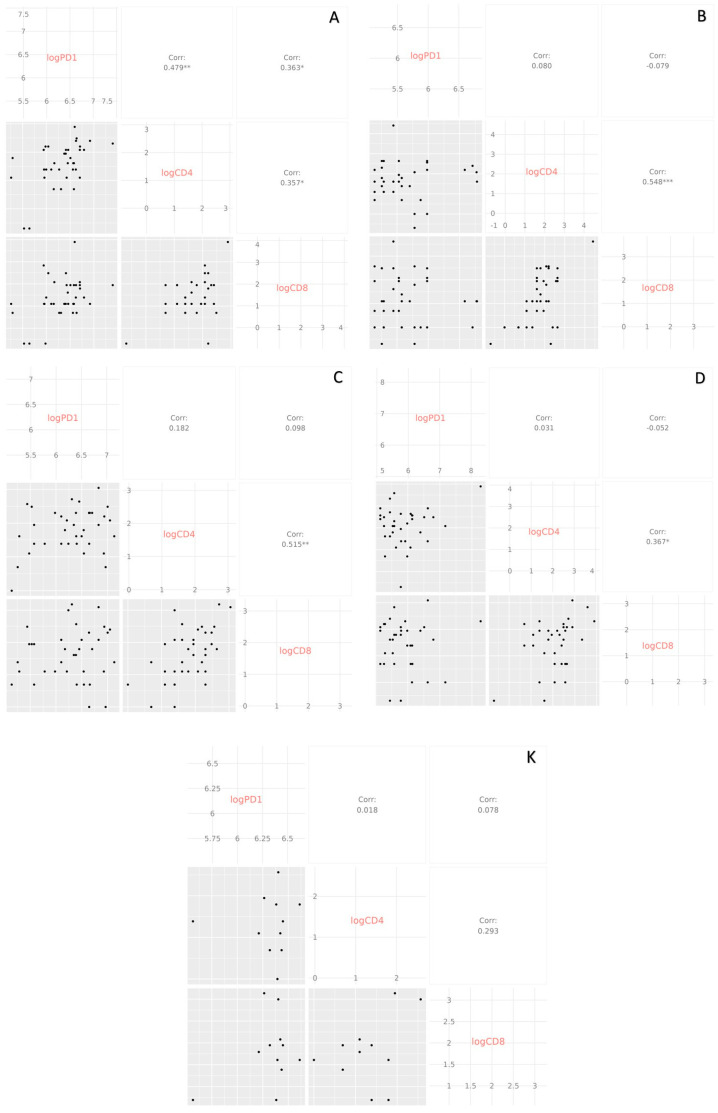
Spearman correlation between sPD-1, CD4^+^ PD-1, and CD8^+^ PD-1, analysed separately for all COVID-19 groups and control subjects. (A) In Group A a positive correlation is observed between sPD-1 with CD4^+^ PD-1 (*p* = 0.01), sPD-1 with CD8^+^ PD-1 (*p* = 0.05) and CD4^+^ PD-1 with CD8^+^ PD-1 (*p* = 0.05). (B) In Group B a positive correlation is observed between CD4^+^ PD-1 and CD8^+^ PD-1 (*p* = 0.001). (C) In Group C a positive correlation is observed between CD4^+^ PD-1 and CD8^+^ PD-1 (*p* = 0.01). (D) In Group D a positive correlation is observed between CD4^+^ PD-1 and CD8^+^ PD-1 (*p* = 0.05). (K) In Group K no positive correlation is observed between sPD-1, CD4^+^ PD-1, and CD8^+^ PD-1. * *p* < 0.05; ** *p* < 0.01; *** *p* < 0.001.

When analysed in detail, a positive correlation between CD4^+^ PD-1 and CD8^+^ PD-1 was found in all COVID-19 groups but not in the control group (Figure 10). Correlations of sPD-1 with CD4^+^ PD-1 (*p* = 0.01) and sPD-1 with CD8^+^ PD-1 (*p* = 0.05) were only observed in patients of Group A. Overall, these findings suggest that plasma PD-1 levels do not correlate with PD-1 expression on T cells. However, there is a significant correlation between CD4^+^ PD-1 and CD8^+^ PD-1 in the patients with COVID-19.

## 4. Discussion

The identification and logical interpretation of selected laboratory parameters is a key step in the stratification of patients with various diseases. In addition to clinical characteristics, a wide range of laboratory parameters—biochemical, haematological, and immunological—are used to identify patients at a high risk for severe COVID-19 [4].

Worldwide studies have shown that SARS-CoV-2 strongly modulates the immune response, leading to a potentially life-threatening systemic delivery of pro-inflammatory cytokines [6,7]. One of the most frequently observed changes in the immune profile of patients with COVID-19 is significant lymphocytopenia, a characteristic feature of severe infection [8,9,10,11], and has been proposed as a predictive biomarker for the severity of COVID-19 [12,13].

In line with published results, we observed a decreasing trend in all immune parameters, including the IRI, with increased disease severity. We also evaluated the temporal evolution and differences in the estimated marginal means of immunological variables after 1 week of hospitalisation in the COVID-19 groups, as well as between groups and between survivors and non-survivors. In general, the total number of all lymphocyte subsets (CD3^+^, CD4^+^, CD8^+^, and CD19^+^) increased across all COVID-19 groups after 1 week of hospitalisation. However, there was no difference in PD-1 and TIM-3 expression over time. Both the decrease in absolute T cell count and the increased expression of immune checkpoint inhibitors could potentially contribute to disease progression and adverse clinical outcomes [11]. In our study, a significantly higher expression of PD-1 on CD4^+^ cells was observed upon hospital admission in non-survivors than in survivors, but no significant difference in PD-1 expression was found on CD8^+^ cells between these groups. Moreover, no significant difference in PD-1 expression on CD4^+^ or CD8^+^ cells was observed when comparing healthy controls with COVID-19 groups, which may have been influenced by the small sample size of the control group. During the first week of hospitalisation, there was no significant change in PD-1 expression. We observed a significantly higher expression of TIM-3 on CD4^+^ and CD8^+^ cells in all COVID-19 groups than in healthy controls; however, only a non-significant trend toward a higher expression of TIM-3 on CD4^+^ and CD8^+^ cells was observed in non-survivors compared to survivors. The expression of TIM-3 remained elevated during the first week of hospitalisation.

Similarly, in 2020, Wang et al. [14] described a significant increase in PD-1 expression on CD4^+^ and CD8^+^ T cells in critically ill individuals, while only a non-significant increasing trend was observed for TIM-3 expression, correlating with disease severity. Compared to healthy controls, Diao et al. [11] reported increased PD-1 expression on both CD4^+^ and CD8^+^ T cells, as well as increased TIM-3 expression on CD4^+^ T cells. A significantly higher proportion of PD-1 was detected in patients who succumbed to the disease than in survivors [11]. Kuri-Cervantes et al. [15] suggested that an increased proportion of PD-1-expressing CD4^+^ T cells is one of the most characteristic findings in patients with severe COVID-19. Furthermore, upregulation of PD-1 on T cells was observed during the progression of symptomatic stages of COVID-19 [11]. In 2022, Beserra et al. [16] reported no difference in PD-1 expression on CD8^+^ T cells. However, one study suggested that CD8^+^ T cells expressing PD-1 may not show dysfunction in the acute phase of the disease, indicating that PD-1 expression in these cells is related to activation rather than the exhaustion model of T lymphocytes in patients with COVID-19 [17].

Our preliminary study described a significantly increased expression of PD-1 on CD4^+^ and CD8^+^ T cells in non-survivors compared to survivors. However, no significant differences in TIM-3 expression were observed between patient groups, suggesting the potential reversibility of immune paralysis in the most severe cases. During clinical recovery, a significant decrease in TIM-3 expression on CD4^+^ and CD8^+^ T cells was observed [18].

According to Martin-Quirós et al. [19], TIM-3 expression on T cells could serve as a prognostic biomarker for patients at high risk for developing secondary bacterial infections and eventual sepsis.

In addition to the membrane-bound checkpoint inhibitors, the concentration of their soluble isoforms should also be considered. These isoforms—produced through membrane cleavage or alternative mRNA splicing—play a role in the competitive regulation of their membrane-bound counterparts [20].

PD-1/PD-L1 proteins are important immunoregulatory markers described in various pathologies [21]. To date, only a few studies have investigated sPD-1 in infectious diseases such as COVID-19 [16,22]. In 2022, Beserra et al. [16] analysed sPD-1 and sPD-L1 in the serum of patients with COVID-19 and found similar levels of sPD-1 across groups, while sPD-L1 levels were elevated in patients with severe and critical symptoms relative to healthy controls, suggesting a relationship between sPD-L1 and COVID-19 severity. Moreover, no changes in sPD-1 or sPD-L1 levels were observed over time following a SARS-CoV-2 diagnosis [16]. A Chinese study further investigated the relationship between soluble checkpoint molecules and COVID-19 progression, reporting higher concentrations of 11 molecules (sGITR, s4-1BB, sTIM-3, sCD27, sLAG-3, sPD-1, sCD28, sCTLA-4, sBTLA, sHVEM, and sCD80) in critical patients than in moderate cases. The study also showed similar dynamic patterns between soluble and membrane-bound counterparts in six patients with critical COVID-19, and flow cytometry revealed higher PD-1 expression on CD4^+^ and CD8^+^ T cells in patients with critical disease in compared to moderate state of disease. However, its small sample size limited the conclusions that could be drawn about the association between soluble and cellular checkpoint molecules [22]. In 2020, Gibellini et al. [23] reported increased concentrations of sPD-1 and sPD-L1 in COVID-19-positive patients compared to controls, with higher levels correlating with disease severity [24]. Conversely, a recent study suggests that higher serum sPD-L1 levels have a protective role in acute respiratory distress syndrome associated with COVID-19. Specifically, sPD-L1 administration alleviated lung inflammation and improved survival in mice, indicating its potential as a therapeutic agent in COVID-19-related acute respiratory distress syndrome [25]. However, the link between PD-1 and poor prognosis in various diseases remains unproven [26]. In our study, we observed similar sPD-1 levels in the control cohort, Group A, and Group C, with lower levels in Groups B and D. When comparing survivors and non-survivors, sPD-1 concentrations were higher in survivors; these unclear results prevent us from considering sPD-1 as a predictive biomarker for severe COVID-19. We hypothesise that sPD-1 may reflect disease progression and immunopathophysiological mechanisms. sPD-1 levels were lower in Group B, in which the disease did not progress, than in Group C, in which critical events occurred, possibly independent of sPD-1. The concentration of sPD-1 detected in Group D may indicate excessive immune hyperactivation and cytokine release leading to death, while the low sPD-1 levels in Group B could predict the insufficient inhibition of immune overactivation. To address this, it is essential to analyse sPD-L1; analysing sPD-1 alone does not provide a comprehensive model of the sPD-1/PD-L1 axis, resulting in unclear immune response interpretations.

In further analyses, we found a statistically significant correlation between the expression of CD4^+^ PD-1 and CD8^+^ PD-1 across all examined groups. Interestingly, a correlation between sPD-1 and CD4^+^ or CD8^+^ PD-1 was observed only in patients with mild-to-moderate disease (Group A). However, no significant correlation between the soluble and membrane-bound forms of PD-1 was detected in the other COVID-19 groups. Based on these results, the correlation between CD4^+^ and CD8^+^ PD-1 was not consistently observed across all analysed groups. We hypothesise that the expression of membrane-bound PD-1 and soluble PD-1 may be driven by different underlying mechanisms.

In our study, we used an imbalanced RF machine-learning algorithm to identify suitable predictive markers of a severe course of COVID-19. Predictors were ranked using VIMP, minimal graph depth, and the elastic net algorithm. However, none of these three ML algorithms provided a clear and consistent ranking of predictors that could reliably predict COVID-19 mortality. We determined the AUC values for all immune parameters—leucocytes/nL, lymphocytes/nL, CD3^+^ %, CD3^+^ cells/µL, CD19^+^ %, CD19^+^ cells/µL, CD4^+^ %, CD4^+^ cells/µL, CD8^+^ %, CD8^+^ cells/µL, NK %, NK cells/µL, IRI, PD-1 CD4^+^ %, PD-1 CD8^+^ %, TIM-3 CD4^+^ %, and TIM-3 CD8^+^ %—and achieved approximately 70% sensitivity and 70% specificity with an AUC value of 0.729 for predicting COVID-19 mortality. Notably, AUC values of >0.80 are generally considered clinically useful, while those <0.80 are of limited clinical value. In 2020, Yadaw et al. [27] evaluated four machine-learning algorithms for predicting COVID-19 mortality, with the XGBoost model achieving the highest performance (AUC = 0.91). A retrospective analysis of 2520 hospitalised patients with COVID-19 showed that a neural network model outperformed the others, achieving an AUC of 0.97 for predicting physiological deterioration and death [28]. In 2022, Moulaei et al. [29] also predicted COVID-19 mortality using data acquisition techniques, concluding that the RF model was the best for predicting mortality, with exceptional performance across metrics, including ROC (1.00), precision (99.74%), specificity (99.84%), and sensitivity (98.25%). One key obstacle in ML algorithms is the issue of imbalanced data, where classes are unequally represented, which often lead to biased results favouring the predominant class. This imbalance is evident in our dataset, where more outcomes were assessed in survivors than in non-survivors, making ML models more likely to categorise new observations in the majority class.

The limitations of this study include its retrospective clinical observational design, unbalanced group size, absence of a non-hospitalised group of patients with COVID-19, and a small control group compared to the COVID-19 group. Additionally, the variable time between the onset of infection and hospital admission might have affected the results. Many findings require further research, particularly in the case of inhibitory molecules, but could hold promise for the targeted treatment of COVID-19.

## 5. Conclusions

The immune system is one of the primary regulatory systems in the human body and plays a crucial role in ongoing SARS-CoV-2 infection. Analyses of the kinetics of lymphocyte populations and subpopulations may serve as potential biomarkers for the early detection of a severe disease course, which is associated with the development of complications and organ damage. High expression of immune checkpoint molecules (e.g., PD-1, TIM-3) has been observed in patients at high risk for a severe disease course. These findings highlight the need for further study to precisely define the role of immune checkpoint molecules in the immune response to SARS-CoV-2.

## Figures and Tables

**Table 1:** Characteristics of the patients included in the COVID-19 study.

Group	Quantity	Age (Years)	Hospitalisation (Days)	Female	Male
K				25	5
	Mean	43.8	0	83.3%	16.7%
	SD	13.6	0		
	Median	44	0		
A				51	49
	Mean	64.2	8.6	51.0%	49.0%
	SD	13.6	5.33		
	Median	67	7		
B				168	210
	Mean	64.2	10.7	44.4%	55.6%
	SD	14.4	5.7		
	Median	65	9		
C				30	60
	Mean	61.4	21.7	33.3%	66.7%
	SD	13.7	13.4		
	Median	63.5	18.1		
D				86	115
	Mean	75.8	12.3	42.8%	57.2%
	SD	11.1	8.1		
	Median	76	10.1		

**Table 2:** Clinical characteristics and therapeutic approaches of the patients included in the COVID-19 study.

	A (n = 100)	B (n = 378)	C (n = 90)	D (n = 201)
Chronic ischaemic heart disease	30 (30.0%)	156 (41.3%)	23 (25.6%)	139 (69.2%)
Hypertension	62 (62.0%)	265 (70.1%)	63 (70.0%)	174 (86.6%)
Obesity	17 (17.0%)	135 (35.7%)	42 (46.7%)	58 (28.8%)
Arrhythmia	12 (12.0%)	65 (17.2%)	6 (6.7%)	57 (28.3%)
Chronic obstructive pulmonary disease	8 (8.0%)	39 (10.3%)	8 (8.9%)	28 (13.9%)
Stroke history	6 (6.0%)	22 (5.8%)	9 (10.0%)	38 (18.9%)
Cancer	12 (12.0%)	16 (4.2%)	5 (5.6%)	25 (12.4%)
Immunodeficiency	1 (1.0%)	1 (0.26%)	5 (5.6%)	3 (1.5%)
Pregnancy	0 (0.0%)	2 (0.52%)	3 (3.3%)	0 (0.0%)
Systemic corticosteroids	42 (42.0%)	339 (89.7%)	86 (95.6%)	172 (85.6%)
Antivirals (remdesivir, favipiravir)	10 (10.0%)	141 (37.3%)	38 (42.2%)	68 (33.8%)

**Table 3:** Clinical characteristics of the patients (Groups A, B, C, and D) and controls (Group K) included in the analysis of soluble PD-1.

Group	Quantity	N	Age (Years)	Female	Male
K		11		4 (36.0%)	7 (63.0%)
	Mean		41		
	SD		11.89		
	Median		38		
A					
	Mean	36	63.61	18 (50.0%)	18 (50.0%)
	SD		14.88		
	Median		63.5		
B					
	Mean	38	66.31	19 (50.0%)	19 (50.0%)
	SD		14.61		
	Median		69.5		
C		36			
	Mean		63.97	11 (30.6%)	25 (69.4%)
	SD		12.42		
	Median		64		
D		35			
	Mean		77.37	13 (37.1%)	22 (62.9%)
	SD		10.81		
	Median		77		

**Table 4:** The estimated marginal means of inhibitory molecules PD-1 and TIM-3 (%) in all analysed COVID-19 groups and the control group upon hospital admission.

Group	PD-1 CD4^+^	PD-1 CD8^+^	TIM-3 CD4^+^	TIM-3 CD8^+^
K	7.5	6.6	56.7	59.2
A	5.9	4.9	69.2	70.6
B	7.0	5.2	69.6	72.1
C	7.2	5.6	75.0	76.6
D	8.4	5.2	71.9	74.1

**Table 5:** Significant post hoc pairwise comparisons between Groups K and D for PD-1 and TIM-3 on CD4^+^ and CD8^+^ T cells (%), showing marginal mean differences (estimates) with lower and upper 95% confidence intervals (CI) and *p* values.

Variable	Contrast	Estimate	*p* Value	Lower CI	Upper CI
PD-1 CD4^+^	A vs. D	−2.441	0.003	−4.310	−0.571
TIM-3 CD4^+^	K vs. A	−12.472	0.006	−22.393	−2.550
	K vs. B	−12.890	0.002	−22.391	−3.389
	K vs. C	−18.369	0.000	−29.278	−7.460
	K vs. D	−15.295	0.001	−25.572	−5.017
	B vs. C	−5.479	0.039	−10.788	−0.171
TIM-3 CD8^+^	K vs. A	−11.151	0.022	−21.270	−1.032
	K vs. B	−12.710	0.003	−22.369	−3.051
	K vs. C	−17.250	0.000	−28.363	−6.137
	K vs. D	−14.681	0.001	−25.134	−4.227

**Table 6:** Estimated marginal means of soluble PD-1 concentration upon hospital admission for all analysed COVID-19 groups.

Parameters	K	A	B	C	D
sPD-1 [pg/mL]	567.6	610.1	317.4	613.1	490.2

## Data Availability

The raw data supporting the conclusions of this article will be made available by the authors upon reasonable request, without undue reservation.

## Supplementary Materials

The following supporting information can be downloaded at: https://www.mdpi.com/article/10.3390/medicina61020189/s1. Table S1. Exploratory data analysis of immune parameters summarized by mean, standard deviation and median in all analysed COVID-19 groups on admission to the hospital. Table S2. Characteristics of the groups and exploratory data analysis of immune parameters and summarized by mean, standard deviation and median between survivors (A, B, C) and non-survivors (D) on admission to the hospital. Table S3. Characteristics of the groups and exploratory data of difference (time 0 − time 1) immune parameters summarized by mean, standard deviation and median in all analysed COVID-19 groups after one week of hospitalization. Table S4. Exploratory data analysis summarized by mean, standard deviation and median of sPD-1 (pg/mL) and PD-1 expressed on CD4^+^ and CD8^+^ analysed in the same patients on admission.
