# Supplementary material for: Lymphocyte Inhibition Mechanisms and Immune Checkpoints in COVID-19: Insights into Prognostic Markers and Disease Severity
Source: Medicina (Kaunas). 2025 Jan 22;61(2):189. doi: 10.3390/medicina61020189 (PMC11857393; doi:10.3390/medicina61020189)
Supplement: Supplementary file 1 [file medicina-61-00189-s001.zip › medicina-3415780-supplementary.pdf]

Table S1. Exploratory data analysis of immune parameters summarized by mean, standard deviation and median in all analysed COVID-19 groups on admission to the hospital.

Group	K				A				B				C				D			
Variable	N	Mean	SD	Median	N	Mean	SD	Median	N	Mean	SD	Median	N	Mean	SD	Median	N	Mean	SD	Median
Leukocytes cells/ul	30	5.793	1.426	5.6	100	7.199	3.744	6.5	378	7.451	3.361	6.9	90	9.752	4.893	8.55	201	9.234	5.9	8.3
Lymphocytes cells/ul	30	1.999	0.652	1.815	100	1.548	0.783	1.525	378	1.133	0.66	0.97	90	1.008	0.598	0.795	201	0.899	0.849	0.72
CD3 %	30	76.7	6.66	76	100	71.36	11.352	73	378	67.225	12.094	69	90	66.711	12.678	68	201	64.891	15.938	66
CD3 cells/µl	30	1418.1	367.178	1369.5	100	1101.06	636.374	992	378	725.886	424.859	656	90	654.478	454.521	551.5	201	565.622	461.701	459
CD19 %	30	9.5	4.826	8	100	11.55	9.037	10	378	13.413	8.184	12	90	15.356	8.488	14	201	12.99	13.18	10
CD19 cells/µl	30	181.033	118.132	155	100	180.06	237.176	125.5	378	149.283	142.919	111	90	138.233	100.951	121.5	201	203.97	1021.834	62
CD4 %	30	49.667	8.044	51	100	45.75	11.622	45	378	42.439	11.611	43	90	44.622	12.007	45	201	38.652	13.622	37
CD4 cells/µl	30	895.667	314.006	839	100	710.48	453.159	630	378	458.825	277.695	420	90	437.689	319.465	357	201	326.204	238.469	274
CD8 %	30	23.133	5.806	23	100	20.78	9.627	20	378	19.963	8.465	18	90	17.844	7.762	17	201	21.687	12.081	20
CD8 cells/µl	30	431.3	162.072	388.5	100	310.23	245.609	253	378	217.275	166.786	177.5	90	175.444	135.623	146	201	201.537	239.511	138
NK %	30	12.6	6.595	12	100	16.9	8.345	14.5	378	18.008	10.556	16	90	16.811	11.878	12	201	20.677	11.985	18
NK cells/µl	30	238.433	145.018	219	100	216.65	129.792	182.5	378	181.235	143.164	149	90	152.144	129.044	125.5	201	183.159	172.341	143
IRI	30	2.33	0.794	2.3	100	2.889	1.934	2.47	378	2.701	1.767	2.38	90	3.181	1.738	2.72	200	2.758	2.693	1.97
PD-1 CD4 %	30	6.433	2.431	6	97	7.887	6.586	6	355	9.372	9.571	7	87	8.908	10.376	7	189	12.836	13.493	9
PD-1 CD8 %	30	7.667	5.628	5	97	7.1	7.145	5	355	7.304	6.333	6	87	8.632	11.301	6	189	8.36	11.212	5
TIM-3 CD4 %	30	53.167	8.694	52.5	61	66.885	15.245	69	206	67.146	16.454	70.5	51	72.98	12.791	74	105	71.029	15.64	75
TIM-3 CD8 %	30	56.333	7.906	57	61	67.836	16.799	72	206	69.369	17.52	73.5	51	74.529	11.707	76	105	72.905	16.333	76

Table S2. Characteristics of the groups and exploratory data analysis of immune parameters and summarized by mean, standard deviation and median between survivors (A, B, C) and non-survivors (D) on admission to the hospital.

Group	ABC				D			
Variable	N	Mean	SD	Median	N	Mean	SD	Median
Leukocytes cells/ul	568	7.771	3.803	7.15	201	9.234	5.9	8.3
Lymphocytes cells/ul	568	1.186	0.695	1.1	201	0.899	0.849	0.72
CD3 %	568	67.871	12.15	70	201	64.891	15.938	66
CD3 cells/µl	568	780.623	495.77	689	201	565.622	461.701	459
CD19 %	568	13.394	8.442	12	201	13	13.17	10
CD19 cells/µl	568	152.952	158.667	118	201	203.98	1021.832	62
CD4 %	568	43.368	11.734	44	201	38.652	13.622	37
CD4 cells/µl	568	499.782	335.721	441.5	201	326.204	238.469	274
CD8 %	568	19.771	8.607	18	201	21.687	12.081	20
CD8 cells/µl	568	227.012	183.364	186.5	201	201.537	239.511	138
NK %	568	17.481	10.44	15	201	20.677	11.985	18
NK cells/µl	568	182.861	139.771	150	201	183.159	172.341	143
IRI	568	2.81	1.798	2.465	200	2.758	2.693	1.97
PD-1 CD4 %	539	9.036	9.243	7	189	12.839	13.491	9
PD-1 CD8 %	539	7.483	7.479	6	189	8.37	11.204	5
TIM-3 CD4 %	318	68.031	15.798	71	105	71.029	15.64	75
TIM-3 CD8 %	318	69.903	16.673	74	105	72.905	16.333	76
CD3 %	568	63.831	14.138	65	201	75.861	11.111	76
Length_hospital	568	12.072	8.582	9.5	201	12.308	8.176	10
Gender	568				201			
Female	249	43.8%			86	42.8%		
Male	319	56.2%			115	57.2%		

Table S3. Characteristics of the groups and exploratory data of difference (time 0 – time 1) immune parameters summarized by mean, standard deviation and median in all analysed COVID-19 groups after one week of hospitalization.

Group	A				B				C				D			
Variable	N	Mean	SD	Median	N	Mean	SD	Median	N	Mean	SD	Median	N	Mean	SD	Median
Leukocytes cells/ul	12	-0.75	3.174	-0.95	88	-1.427	3.195	-1.25	31	-1.535	8.084	-0.3	27	-5.204	7.876	-4.1
Lymphocytes cells/ul	12	-0.381	0.519	-0.395	88	-0.742	0.84	-0.605	32	-0.426	1.023	-0.29	27	-1.269	6.159	-0.01
CD3 %	12	-3	9.391	-0.5	88	-5.943	10.741	-5	32	-3.04	16.356	-5	27	-3.63	12.92	-3
CD3 cells/µl	12	-227.33	526.515	-299.5	88	-558.716	692.539	-412.5	32	-274.406	761.96	-228.5	27	-278.556	1168.831	-68
CD19 %	12	-2.333	5.193	-3.5	88	-1.034	8.757	-1	32	-30.969	165.879	-2	27	-5.037	9.023	-2
CD19 cells/µl	12	-91.917	91.776	-77	88	-127.239	243.917	-75.5	32	-80.156	194.796	-53	27	-932.63	4496.966	-29
CD4 %	12	-6.833	11.199	-4.5	88	-7.25	10.486	-7	32	-4.25	10.761	-6.5	27	-3.852	10.719	-3
CD4 cells/µl	12	-262.75	320.989	-278	88	-414.875	433.132	-341.5	32	-211.375	578.726	-170.5	27	-246.296	822.324	-80
CD8 %	12	2.5	4.964	2	88	-1.114	11.488	0	32	-10.719	55.401	-0.5	27	0.37	7.967	0
CD8 cells/µl	12	-27.583	160.29	-73.5	88	-123.591	239.547	-98	32	-59.969	202.47	-75.5	27	-37.593	377.978	-9
NK %	12	5	8.29	3	88	6.5	9.112	4.5	32	-0.5	44.412	6.5	27	9.111	13.279	4
NK cells/µl	12	24.5	169.009	2	88	-1.364	143.942	-8	32	47.094	134.548	17	27	44.037	227.691	48
IRI	12	-0.812	1.422	-0.555	88	-0.379	1.527	-0.235	32	-8.574	48.113	-0.195	27	-0.385	4.12	-0.08
PD-1 CD4 %	11	-0.909	17.155	3	81	-1.568	8.139	-1	30	-3.073	14.9	0	22	-1.227	10.51	0
PD-1 CD8 %	11	2.909	14.909	0	81	-1	8.323	-1	30	-2.5	7.794	-1.5	22	-1.182	8.122	0
TIM-3 CD4 %	5	-4.4	7.537	-3	45	-2.067	20.158	-2	13	5.385	19.47	8	11	6.818	31.622	0
TIM-3 CD8 %	5	-4	7.969	-1	45	-0.867	17.657	0	13	8.308	21.914	11	11	5.364	36.388	0
Age	12	70.667	14.022	69.5	88	69.375	12.103	70	32	65.5	11.857	64.5	27	72.481	12.22	72
Length_hospital	12	14.667	6.692	13	88	14.875	6.216	14	32	26.188	13.82	22.5	27	18.593	8.984	19
Gender	12				88				32				27			
Female	7	58.3%			38	43.2%			14	43.8%			13	48.1%		
Male	5	41.7%			50	56.8%			18	56.2%			14	51.9%		

Table S4. Exploratory data analysis summarized by mean, standard deviation and median of sPD-1 (pg/ml) and PD-1 expressed on $CD4^+$ and $CD8^+$ analysed in the same patients on admission.

Group	K				A				B				C				D			
Variable	N	Mean	SD	Median	N	Mean	SD	Median	N	Mean	SD	Median	N	Mean	SD	Median	N	Mean	SD	Median
sPD-1 pg/ml	11	567.636	119.99	598	36	610.16	265.77	613.5	38	317.44	189.66	252	36	613.16	303.164	595.5	35	490.28	641.27	324
$CD4^+$ PD-1 %	11	4.636	3.35	4	36	6.02	3.69	5	38	8.34	12.88	5.5	36	7.27	4.32	6.5	35	11.17	9.76	9
$CD8^+$ PD-1 %	11	8.091	6.93	6	36	5.94	8.20	3	38	5.47	6.64	3	36	6.91	5.699	5.5	35	5.82	4.62	5
